# Neuroscience in peripheral cancers: tumors hijacking nerves and neuroimmune crosstalk

**DOI:** 10.1002/mco2.784

**Published:** 2024-10-31

**Authors:** Hua‐Yang Fan, Xin‐Hua Liang, Ya‐Ling Tang

**Affiliations:** ^1^ State Key Laboratory of Oral Diseases, National Center for Stomatology, National Clinical Research Center for Oral Diseases, Department of Oral and Maxillofacial Surgery West China Hospital of Stomatology Sichuan University Chengdu China; ^2^ State Key Laboratory of Oral Diseases, National Center for Stomatology, National Clinical Research Center for Oral Diseases, Department of Oral Pathology West China Hospital of Stomatology Sichuan University Chengdu China

**Keywords:** cancer neuroscience, cancer therapy, neuroimmune crosstalk, peripheral nervous system, tumor microenvironment, tumor‒nerve interactions

## Abstract

Cancer neuroscience is an emerging field that investigates the intricate relationship between the nervous system and cancer, gaining increasing recognition for its importance. The central nervous system governs the development of the nervous system and directly affects brain tumors, and the peripheral nervous system (PNS) shapes the tumor microenvironment (TME) of peripheral tumors. Both systems are crucial in cancer initiation and progression, with recent studies revealing a more intricate role of the PNS within the TME. Tumors not only invade nerves but also persuade them through remodeling to further promote malignancy, creating a bidirectional interaction between nerves and cancers. Notably, immune cells also contribute to this communication, forming a triangular relationship that influences protumor inflammation and the effectiveness of immunotherapy. This review delves into the intricate mechanisms connecting the PNS and tumors, focusing on how various immune cell types influence nerve‒tumor interactions, emphasizing the clinical relevance of nerve‒tumor and nerve‒immune dynamics. By deepening our understanding of the interplay between nerves, cancer, and immune cells, this review has the potential to reshape tumor biology insights, inspire innovative therapies, and improve clinical outcomes for cancer patients.

## Graphical abstract

The figure depicts the intricate interactions between cancer cells, the immune system, and nerves within the tumor microenvironment. At the center, the diagram highlights the tripartite relationship among cancer, immune, and nerve cells, illustrating the dynamic and reciprocal influences they exert on one another. Surrounding this core, key biological processes are illustrated, including proliferation and apoptosis, cell cycle arrest and DNA damage, invasion and metastasis, angiogenesis and lymphangiogenesis, and immune modulation. The diagram also underscores the feedback loop driving cancer progression, where nerves facilitate cancer initiation and immune evasion, while cancer cells activate neural pathways, further enhancing their invasive potential.

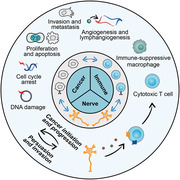

## 1 INTRODUCTION

The nervous system is crucial for organ development, tissue homeostasis, and cancer regulation, as recent research extensively explores the connection between nerves and cancer, thereby establishing a solid foundation for cancer neuroscience.^1^, ^2^, ^3^ The central nervous system (CNS) is directly involved in the formation of brain tumors, with its development closely correlated with tumor progression.^4^ Alterations in different brain regions or neural pathways are intertwined, and abnormal signals originating from brain regions can also modulate peripheral tumor development through the PNS.^5^ Numerous studies have demonstrated that the PNS significantly contributes to the pathogenesis of extracerebral cancers.

The PNS consists of afferent (sensory) and efferent (motor and autonomic) nerves, which branch throughout the body alongside microvessels, connecting the CNS to organs and tissues to maintain local homeostasis.^6^ Over a century ago, pathologists recognized the relationship between peripheral nerves and malignant cells, termed perineural infiltration (PNI), which is associated with increased cancer aggressiveness and poor prognosis.^7^, ^8^ Additionally, researchers have observed a notable increase in nerve density as precancerous lesions progress to cancer.^9^, ^10^ Tumors not only invade nerves but also accelerate cancer progression by secreting molecules that promote nerve infiltration.^11^ For example, neurotrophic factors such as nerve growth factor (NGF) stimulate nerve growth in the tumor microenvironment (TME), leading to sensory and sympathetic nerve infiltration.^12^, ^13^ Cancer cells also release axon guidance molecules, such as semaphorins,^14^, ^15^ that reprogram sensory nerves into sympathetic ones, further driving tumor invasion and metastasis. This interplay underscores the critical role of nerves in promoting tumor aggressiveness. Emerging evidence reveals the intricate interactions between immune cells, cancer cells, and nerves within the TME.^16^ Besides tumor cells, immune cells—such as T cells, myeloid‐derived suppressor cells (MDSCs), and tumor‐associated macrophages (TAMs)—express neural signaling receptors, including adrenergic and cholinergic receptors. These neural signals regulate the movement, proliferation, activation, and function of immune cells, influencing tumor inflammation, anti‐tumor immunity, and immunosuppression.^17^, ^18^ Thus, the peripheral neural network is now recognized as a key component of the TME, contributing to cancer progression, proliferation, invasion, and metastasis through via neural signaling pathways.^19^, ^20^, ^21^, ^22^

The interplay between the nervous system and tumors presents promising therapeutic opportunities, such as inhibiting tumor growth,^23^, ^24^, ^25^ overcoming treatment resistance,^26^, ^27^, ^28^ enhancing immune responses,^29^, ^30^ and preventing metastasis.^31^ Modulating adrenergic and cholinergic signaling can influence these aspects of cancer biology. β‐adrenergic receptor (β‐AR) blockers and parasympathomimetics show potential, especially when combined with chemotherapy or immunotherapy, to enhance treatment efficacy and reduce resistance.^32^, ^33^, ^34^, ^35^, ^36^, ^37^ Notably, clinical trials have demonstrated the effectiveness of combining β‐AR blockers with immune checkpoint inhibitors (ICIs) in treating melanoma.^38^, ^39^, ^40^ Nonetheless, due to the variability in response among different cancer types to therapies targeting nerve‒tumor‒immune interactions, more preclinical trials are necessary for further validation. Targeting neural regeneration pathways, especially those involving NGF, may inhibit tumor progression, as cancers such as pancreatic and breast cancer promote nerve growth to facilitate metastasis.^41^, ^42^, ^43^ Consequently, disrupting these neurogenic processes could offer a strategic approach to limiting metastasis. In conclusion, elucidating the role of the nervous system in cancer progression and its impact on the immune microenvironment is essential for advancing therapeutic strategies and enhancing patient outcomes.

In this review, we outline the interactions between peripheral nerves and cancer, emphasizing the key nerve types and signaling pathways that promote tumor progression. We highlight the bidirectional relationship in which nerves not only regulate tumor initiation and progression but are also hijacked and manipulated by the tumor, creating a self‐sustaining cycle that accelerates tumor development. Additionally, we discuss the role of immune cell phenotypes in nerve‒tumor crosstalk, highlighting their impact on both nerve and tumor dynamics, as well as the molecular mechanisms involved. The review further assesses current clinical treatment strategies and proposes future research directions based on these insights. To our knowledge, it is the first comprehensive review of peripheral nerve‒tumor‒immune crosstalk and its clinical significance.

## 2 NEURAL REGULATION OF THE TME

**FIGURE 1:**
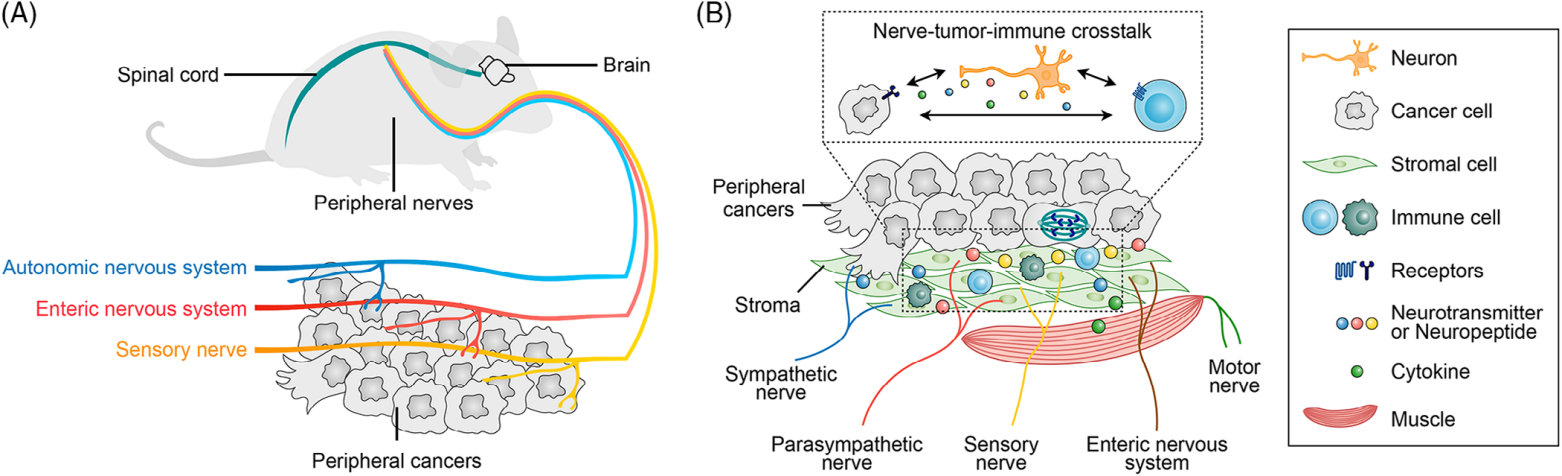
Schematic diagram of peripheral nerves intertwined with peripheral cancers. (A) The sympathetic, parasympathetic, and sensory nerves play a regulatory role in peripheral cancers. (B) This diagram shows how nerves, tumor cells, immune cells, and other components interact within the tumor microenvironment (TME) of peripheral cancers. Various types of nerves—sympathetic, parasympathetic, sensory, motor, and enteric—enter the tissue around tumor cells. The diagram highlights how these nerves communicate with cancer cells by releasing neurotransmitters and neuropeptides, which in turn influence tumor growth and the immune response.

Research has shown that the autonomic nervous system (ANS),^44^, ^45^, ^46^, ^47^, ^48^ sensory nerves,^49^, ^50^ and the enteric nervous system (ENS)^51^, ^52^ play significant roles in the initiation and progression of peripheral tumors (Figure 1A). Denervation studies have demonstrated the direct impact of nerves on tumor growth.^53^, ^54^, ^55^ Neural signals influence by acting on nerve‐related receptors on various cell types within the TME, such as tumor cells and immune cells (Figure 1B). Interestingly, while the connection between physical activity and cancer is well established,^56^, ^57^ the direct involvement of motor nerves in tumor development remains unexplored, warranting further investigation in future research.

### 2.1 Principal nerves involved in peripheral tumors

#### 2.1.1 Autonomic nervous system

The ANS, consisting of the sympathetic and parasympathetic nerves, regulates homeostasis throughout the body.^58^, ^59^, ^60^ The sympathetic (adrenergic) nerves control the “fight or flight” response,^61^, ^62^ while the parasympathetic (cholinergic) nerves manage “rest and digest” functions.^63^, ^64^ These systems maintain physiological balance by exerting opposing effects through the release of neurotransmitters such as norepinephrine and acetylcholine (ACh). Beyond homeostasis, these neurotransmitters are crucial in tumor development by interacting with receptors on tumor and/or immune cells in the TME, directly influencing tumor growth and metastasis.^65^

Adrenergic receptors (ARs) are primarily classified into α and β types,^66^, ^67^ while cholinergic receptors include muscarinic (M) receptors and nicotinic (N) receptors,^68^ both widely distributed on peripheral tumor and immune cells.^69^, ^70^, ^71^, ^72^, ^73^ The α‐ARs have two subtypes, α1 and α2, while β‐ARs consist of β1, β2, and β3. Among these, β2‐AR is the most studied in tumors, present in most cell types, including tumor cells and immune cells.^74^, ^75^, ^76^ Numerous studies have shown that ARs primarily promote tumor growth, and ARs blockers have been used as therapeutic agents for cancers such as prostate,^77^ pancreatic,^44^ gastric,^78^, ^79^ oral,^80^ ovarian,^81^ melanoma,^82^ hepatocellular carcinoma,^83^ high‐grade serous carcinoma,^84^ and breast cancers.^85^, ^86^ Muscarinic receptors, which are G‐protein‐coupled, have five subtypes (M1‒M5),^87^ while nicotinic receptors are ion channel receptors composed of 17 different subunits.^88^ Compared to ARs, cholinergic receptors are less studied in tumors. Activation of M1 and M4 muscarinic receptors can promote the growth and metastasis of prostate cancer cells and contribute to chemotherapy resistance.^89^, ^90^ The M3 muscarinic receptor is highly expressed in colorectal cancer (CRC), with its high expression associated with increased survival rates.^91^ Inhibitors of the M3 receptor can reduce lung cancer growth and immune suppression by inhibiting the AKT and ERK pathways, thereby enhancing anti‐cancer immune responses.^36^ In pancreatic ductal adenocarcinoma (PDAC), the M1 muscarinic receptor signaling pathway suppresses tumor formation, inhibits proliferation, and prolongs survival.^44^ Nicotinic receptors, crucial for rapid excitatory signaling in synaptic transmission, are strongly associated with lung cancer, where they promote proliferation, epithelial‒mesenchymal transition (EMT), angiogenesis, and anti‐apoptotic effects.^92^, ^93^, ^94^ Additionally, tumors can induce the growth of autonomic nerves, creating a feedback loop that increases tumor aggressiveness and supports tumor progression. Both adrenergic and cholinergic receptors are involved in the recruitment, activation, and function of immune cells, such as T cells and TAMs, influencing anti‐tumor immunity and immune exhaustion, thereby affecting tumor progression.^95^, ^96^, ^97^ The complex interactions between the ANS, tumor cells, and immune cells within the TME underscore the significant role of the ANS in cancer biology.

#### 2.1.2 Sensory nerves

Sensory nerves are distributed throughout tissues and organs, including the skin, mucous membranes, muscles, and internal organs, where they detect external stimuli and relay information to the CNS, aiding in homeostasis and responses to environmental changes.^98^ Neuropeptides released by sensory nerves are crucial in pain transmission and inflammatory responses. For example, substance P (SP) amplifies pain signals and induces local inflammation by activating specific receptors, promoting vasodilation, particularly in the trigeminal nervous system.^99^, ^100^ In contrast, somatostatin reduces neuroinflammation and protects nerve function by inhibiting hormone secretion, and the neuropeptide galanin modulates pain and promotes nerve repair.^101^ Together, these neuropeptides act synergistically to regulate pain and inflammation. SP and calcitonin gene‐related peptide (CGRP) are the most extensively studied neuropeptides in the TME, where they interact with tumor cells to stimulate angiogenesis, enhance cancer cell proliferation, and promote metastasis.^102^, ^103^, ^104^, ^105^, ^106^, ^107^, ^108^ The roles of other neuropeptides, such as somatostatin and galanin, in tumors remain largely unexplored, presenting an interesting direction for future exploration in cancer neuroscience. Additionally, sensory nerves influence the TME by affecting immune cells, such as macrophages and lymphocytes, thereby altering inflammatory responses and immune surveillance.^109^, ^110^ Furthermore, interactions between sensory nerves and tumor cells can trigger tumor‐associated pain,^111^, ^112^, ^113^ which reduces the patient's quality of life and may also promote tumor growth and progression.

#### 2.1.3 Enteric nervous system

The ENS consists of enteric neurons and glial cells, often referred to as the “second brain” that functions independently of the ANS.^114^, ^115^ The ENS contains both autonomic and sensory components, expressing various neurotransmitters and neuropeptides that together regulate gastrointestinal function.^116^ Molecules, released by the ENS, can promote cancer cell proliferation, invasion, and resistance to chemotherapy, playing a critical role in the progression of gastrointestinal tumors, particularly colorectal cancer (CRC).^117^, ^118^, ^119^ Studies have demonstrated that increased neural innervation and the severity of neural invasion in CRC, pancreatic cancer, and esophagogastric junction cancer are correlated with reduced overall survival (OS) rates.^120^ Studies have demonstrated that increased neural innervation and the severity of neural invasion in CRC,^10^ pancreatic cancer,^120^, ^121^ and esophagogastric junction cancer are associated with reduced OS rates. In the invasive front of CRC, myenteric plexus neurons are often surrounded by abundant extracellular matrix, accompanied by numerous myelin‐like structures, and significant infiltration of mast cells and plasma cells.^122^ In heterotypic three‐dimensional (3D) culture models, CRC and pancreatic cancer cells are chemotactically attracted to ENS neurons through neurotrophic factors such as NGF and glial cell line‐derived neurotrophic factor (GDNF), migrating along axons via adhesion molecules such as L1 cell adhesion molecule (L1CAM) and N‐cadherin.^123^, ^124^, ^125^ In gastric cancer, doublecortin‐like kinase 1 (DCLK1)^+^ tuft cells and nerves have been identified as the main source of ACh in the mouse gastric mucosa.^21^ These cells promote tumor proliferation and local nerve growth by releasing ACh and upregulating NGF, creating a self‐perpetuating cycle. Complex interactions between gastrointestinal cancers—such as pancreatic and CRCs—and both extrinsic innervation via the vagus nerve and intrinsic ENS innervation contribute to tumor growth.^120^, ^126^, ^127^ Neural inputs enhance tumor growth by upregulating neurotrophic factors, expanding the cancer stem cell population, and modulating tumor‐associated inflammation.^95^ The reciprocal relationship between the ENS and gastrointestinal tumors supports mutual development, with nerves serving as pathways for cancer invasion and neuropeptides from extrinsic nerve terminals promoting further tumor proliferation and invasion.^128^

#### 2.1.4 Motor nerves

Currently, there is a lack of research demonstrating that motor nerves directly influence tumor progression. However, the benefits of exercise for cancer patients are widely recognized.^56^ Regular physical activity has been shown to reduce the risk of developing cancers such as colorectal, breast, and prostate cancer, with relative risk reductions ranging from 10% to 27%.^129^ Exercise has a significant regulatory effect on the TME, particularly in metabolic processes, including specific metabolites such as lactate, tumor signaling pathways, and central carbon metabolism such as the tricarboxylic acid cycle, thereby exhibiting anti‐tumor activity.^130^ Furthermore, exercise modulates the infiltration and function of immune cells within the TME.^131^, ^132^, ^133^ For example, exercise has been shown to inhibit tumor growth in mouse models of breast cancer, induce vascular normalization, promote CD8^+^ T‐cell infiltration, enhance effector functions, and improve the effectiveness of immunotherapy for breast cancer.^134^ Overall, current research suggests that exercise exerts anti‐tumor effects primarily by altering the metabolic and immune characteristics of tumors. Additionally, cytokines or neurotrophic factors produced by motor nerves or skeletal muscles during exercise may have unexpected effects on body cells, warranting further investigation.^135^, ^136^, ^137^ For instance, although interleukin‐6 (IL‐6) is generally recognized for its tumor‐promoting effects, IL‐6 released from skeletal muscle during exercise enhances insulin sensitivity, stimulates the production of anti‐inflammatory cytokines, and reduces proliferation and DNA damage in cancer cells.^129^ GDNF, initially isolated from rat glial cell lines, is closely associated with the PNI in tumor tissues, with motor nerves and skeletal muscles serving as significant sources, and exercise shown to increase its protein levels in tissues.^138^, ^139^ This finding contrasts with the observation that high GDNF levels promote PNI, leading to poorer outcomes for cancer patients. Therefore, exploring the regulatory role of motor nerves in the TME presents an intriguing and promising research area.

### 2.2 Denervation

**FIGURE 2:**
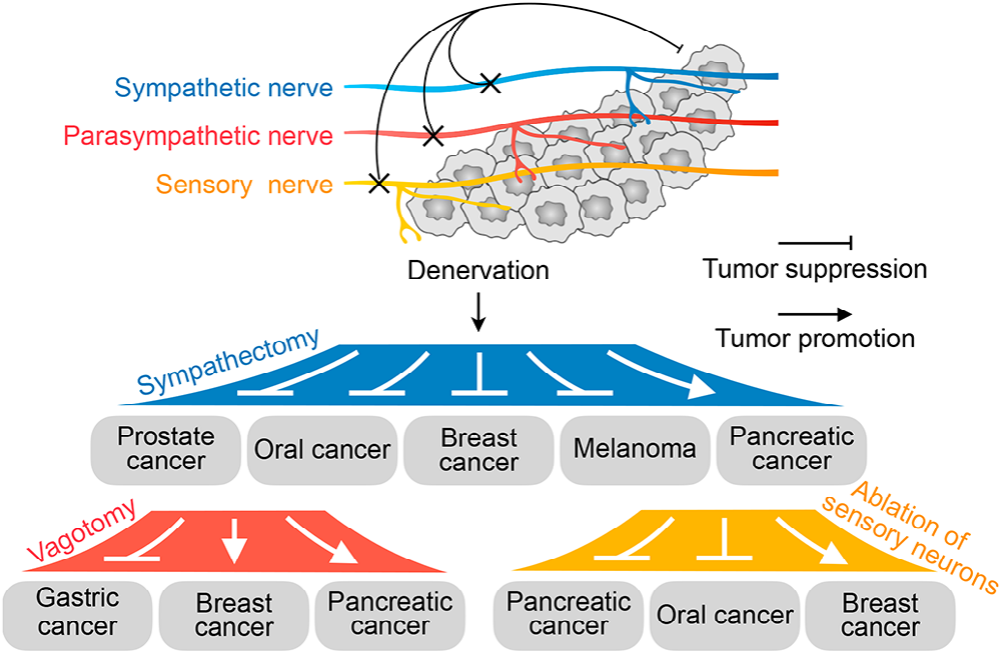
The impact of denervation on peripheral cancers. This diagram illustrates how denervation of different nerve types—sympathetic, parasympathetic, and sensory—affects tumor growth in different peripheral cancers, highlighting the diverse effects based on nerve type and cancer type.

A common approach to investigate the effect of nerves on tumors is to observe tumorigenesis following denervation (Figure 2). Sympathectomy can be achieved through surgical operations or chemical means, such as with 6‐hydroxydopamine (6OHDA).^140^ In both the autochthonous transgenic prostate cancer mouse model and the prostate cancer xenograft mouse model, sympathectomy of bilateral hypogastric nerves inhibited tumor growth and induced tumor regression, respectively.^141^ Sympathectomy of bilateral superior cervical ganglia suppressed tumor growth in 4‐nitroquinoline‐n‐oxide (4‐NQO) induced and orthotopic tongue cancer xenograft mouse models.^142^, ^143^ In addition, sympathectomy can inhibit the growth of breast cancer and melanoma.^144^, ^145^ Sympathetic nerves are also associated with tumor metastasis, and in a mouse model of melanoma, sympathectomy eliminated peak neural activity that is highly correlated with metastasis.^146^ The vagus nerves are mixed nerves that contain both parasympathetic and sensory components and are among the major parasympathetic nerves. The vagus nerve distributed in the gastrointestinal tract is primarily parasympathetic.^147^ Gastric vagotomy attenuated tumor development in three separate gastric cancer mouse models but only in vagotomized tissue.^53^ Similarly, the vagus nerve innervates the pancreas through a parasympathetic component, and unlike its procancer effects in gastric cancer, it has anti‐tumor effects in pancreatic cancer.^126^ In a mouse model of PDAC, vagotomy caused malignant epithelial cell proliferation and elevated pancreatic tumor incidence.^44^ Similar to parasympathetic nerves, sensory nerves may play opposite roles in different tumors. In breast cancer, sensory nerves act as parasympathetic nerves, and disruption of sensory neurons by capsaicin increases cancer metastasis.^148^ The development of PDAC is strongly related to preexisting pancreatitis. Enhanced expression of TRPV1 and TRPA1 channels in sensory neurons might drive inflammation and accelerate precancerous pancreatic cancer through a neurogenic mechanism in a PDAC mouse model.^149^, ^150^, ^151^ Ablating sensory neurons in a mouse model of pancreatitis eliminated inflammation and reduced progression to cancer.^152^ Nociceptors are the nerve endings of sensory neurons that can be involved in regulating immune and inflammatory responses.^153^ Oral squamous cell carcinoma (OSCC) is densely innervated by nociceptive nerves originating from the trigeminal ganglion, and denervation of sensory nerves can inhibit tumor growth.^54^, ^154^ Nociceptive stimuli such as peripheral nerve‐derived neuropeptides, including CGRP and SP, and increased infiltration of αCGRP^+^ sensory nerve fibers were found in OSCC histologic samples.^155^, ^156^ These findings highlight the varying roles of specific types of nerves in different cancer types.

Denervation in PDAC is a multifaceted process shaped by the immunosuppressive TME and the intricate interplay between the nervous system and tumor biology, with the TME being defined by limited effector T‐cell infiltration and a dominance of M2 macrophages and regulatory T (Treg) cells that release anti‐inflammatory factors, thereby suppressing anti‐tumor immune responses and enabling tumor evasion and growth.^157^, ^158^, ^159^ Denervation, particularly the removal of sympathetic nerves, can disrupt neural signals that facilitate tumor progression but also triggers intricate immune and cellular responses. For example, sympathetic nerve removal has been associated with an increase in M2 macrophages, which contribute to immunosuppression and tumor growth by secreting cytokines such as IL‐10 that inhibit T‐cell‐mediated immunity.^160^, ^161^ The interaction between the nervous and immune systems shapes immune activity within the TME and influences tumor behavior, with denervation potentially disrupting these pathways, impairing the function of immune cells, particularly macrophages, while also directly altering the tumor's immune landscape by reducing the anti‐tumor activity of T cells, thereby weakening immune surveillance and promoting tumor growth.^96^, ^162^ Sympathetic nerves also play a critical role in tumor angiogenesis, and their removal may indirectly affect tumor growth and invasion. In summary, denervation in PDAC poses a complex therapeutic challenge, as it may inhibit tumor growth by disrupting neural‐tumor signaling but could also inadvertently promote tumor progression through immune dysregulation and altered angiogenesis, underscoring the need for cautious evaluation of denervation as a treatment strategy to prevent unintended consequences.

### 2.3 Neural signals and channels

Ion channels are membrane proteins that enable ion movement between cellular compartments, a process critical for electrical signaling and cell motility.^163^ These channels help maintain normal tissue homeostasis, and dysregulation of their expression or function can contribute to the transformation of normal cells into cancer cells, characterized by unchecked growth and spread.^164^ Neural signals modulate ion channels on cancer cells, influencing growth, cell death, and migration.^165^, ^166^, ^167^ These signals primarily affect the electrical activity and cell cycle of cancer cells by regulating voltage‐gated potassium and sodium channels.^168^, ^169^ Neurotransmitters such as ACh and norepinephrine can bind to receptors on cancer cells, leading to abnormal ion channel activation; for instance, norepinephrine released by the sympathetic nervous system activates β‐ARs, altering ion channel activity and increasing calcium influx, which promotes cancer cell survival and growth.^92^, ^170^ Additionally, neuropeptides and other signaling molecules from nerve cells can regulate ion channel expression and function in cancer cells. Neuropeptides such as SP and CGRP can influence ion channel activity, impacting cancer cell behavior and contributing to tumor progression.^108^, ^171^ In conclusion, neural signals regulate ion channels on cancer cells by altering the local electrical and chemical environment, thereby influencing tumor growth, invasion, and treatment resistance.

## 3 INTERACTIONS BETWEEN PERIPHERAL TUMORS AND NERVES

Clinical samples consistently show that tumor tissues have a higher density of nerve fibers compared to surrounding normal tissues, indicating that cancer cells may promote nerve fiber growth, thereby increasing the presence of neural structures and terminals within the TME.^172^, ^173^, ^174^ Emerging evidence suggests that tumor growth depends not only on blood vessels formed through angiogenesis but also on nerves generated through neurogenesis. This section explores the composition of nerves within tumors, neural signals, and the complex tumor‐nerve interactions, including the role of neural signals in tumor progression, as well as the invasion and remodeling of nerves by tumors.

### 3.1 How the PNS interferes with cancer

**FIGURE 3:**
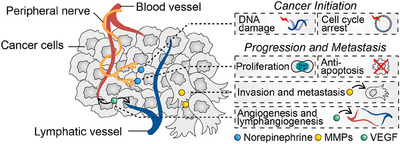
Neural regulation of tumor initiation and progression. Neural signals regulate tumorigenesis, including their effects on cellular DNA damage and the cell cycle, as well as tumor progression, including cell proliferation, anti‐apoptosis, invasion, metastasis, angiogenesis, and lymphangiogenesis.

Research indicates that neural signals in the TME influence every stage of peripheral tumor development, from initiation to invasiveness, while the peripheral nervous system (PNS) impacts cancer progression by affecting DNA damage repair, cell cycle regulation, proliferation, apoptosis resistance, migration, invasion, EMT, angiogenesis, and lymphangiogenesis (Figure 3).^65^, ^175^

#### 3.1.1 Initiation

Research indicates that the intrinsic risks of tumor development include DNA damage responses, cell cycle checkpoint regulation, oxidative stress responses, chromatin structure alterations, and transcription/translation networks, all of which can confer a selective advantage to cells and drive malignant growth.^176^, ^177^, ^178^ Additionally, some tumors are associated with specific environmental factors such as smoking (lung cancer) and sun exposure (skin cancer), as well as endogenous factors such as immune status, metabolism, and hormone levels, collectively defined as non‐intrinsic risk factors.^177^

Neural signals in tumors are influenced by both intrinsic and non‐intrinsic factors. Among the non‐intrinsic factors, environmental influences such as smoking and sun exposure are closely linked to neural signaling pathways. For example, during smoking, nicotine binds to nicotinic ACh receptors, opening ion channels to allow sodium and potassium ion flow, thereby altering neuronal membrane potential and triggering the release of neurotransmitters such as dopamine (DA).^179^, ^180^, ^181^ Similarly, sun exposure has a complex relationship with stress, as the sympathetic nervous system, by releasing catecholamines such as adrenaline and noradrenaline, triggers various physiological responses. This exposure influences the release of stress hormones, which affect hormone levels, metabolism, and immunity, thereby contributing to intrinsic cancer risks.^182^, ^183^ Stress is closely linked to cell cycle regulation and DNA damage, with the sympathetic nervous system releasing noradrenaline, which influences DNA damage/repair and cell cycle progression, potentially promoting cancer initiation.^184^, ^185^, ^186^ In vitro studies have shown that human ovarian cancer cells exhibit accelerated DNA damage when exposed to stress.^186^ Similarly, stress hormones can cause DNA damage in breast cancer cells and induce cell cycle arrest at the G1 phase.^187^ Furthermore, noradrenaline activation of β‐ARs in human osteosarcoma cells leads to DNA damage accumulation and a decrease in tumor suppressor gene p53 levels.^188^, ^189^ In vivo studies have shown that stressed mice treated with noradrenaline exhibit increased DNA damage and reduced DNA repair.^190^ Additionally, cholinergic activation results in the inhibition of p‐ERK1/2 and p‐p38 MAPK in human pancreatic cancer cells, reducing cell viability and invasion both in vitro and in vivo, and inducing cell cycle arrest.^191^ Knockdown of the cholinergic receptor nicotinic α5 (CHRNA5) in breast cancer cells also promotes cell cycle arrest and inhibits DNA synthesis.^192^ It is important to note that tumor development is not solely caused by DNA damage but also requires the accumulation and maintenance of these damages during cell division, along with increased proliferation and anti‐apoptotic capabilities. Moreover, malignant cells must overcome multiple barriers to successfully form tumors, such as evading immune attack and transforming the surrounding stroma into a tumor‐supportive TME.

#### 3.1.2 Progression and metastasis

Neural signals play a critical role in multiple aspects of tumor development, including proliferation, apoptosis, invasion, metastasis, angiogenesis, and lymphangiogenesis. For instance, activation of β2‐adrenergic signaling enhances proliferation in gastric cancer cells,^78^, ^79^ whereas inhibition of this signaling induces apoptosis.^193^ Similarly, in breast cancer cells, β2‐adrenergic signaling drives proliferation by activating the tyrosine kinase effector GSK3.^194^ In ovarian cancer, adrenergic signaling enhances anti‐apoptotic mechanisms by increasing focal adhesion kinase levels.^195^ In pancreatic cancer cells, blocking β2‐ARs leads to G1/S phase cell cycle arrest and induces cell death.^196^ Likewise, selective inhibition of β2‐adrenergic signaling in colorectal cancer cells causes G1 phase cell cycle arrest and triggers apoptosis.^197^ In liver cancer, β2‐adrenergic signaling supports tumor proliferation and survival by downregulating autophagy and stabilizing hypoxia‐inducible factor‐1α.^198^ In gastric epithelium, cholinergic stimulation induces NGF production, promoting tumor formation and growth via the M3 receptor and Yes‐associated protein pathways.^199^ Human gastric cancer cell lines also show overexpression of choline acetyltransferase, and autocrine and paracrine ACh activation of M3 cholinergic receptors, along with downstream epidermal growth factor receptor signaling, stimulates proliferation in these cells.^200^

Neural signals also play a significant role in promoting cancer stemness, invasion, and metastasis. In depressed gastric cancer patients, increased plasma catecholamine levels activate β2‐ARs, leading to the upregulation of MACC1 expression, which enhances cell migration and invasion.^201^ In colorectal cancer, stress‐induced norepinephrine activates β2‐ARs via the CEBPB/TRIM2/P53 axis, promoting EMT.^202^ In a mouse model of prostate cancer, norepinephrine increases lymph node metastasis, an effect that can be blocked by propranolol.^77^ In ovarian cancer cells, norepinephrine boosts prostaglandin E2 synthesis through the NF‐κB‒PTGS2 (nuclear factor kappa‐B and prostaglandin‐endoperoxide synthase 2) pathway, promoting tumor growth and metastasis.^81^ High norepinephrine levels in ovarian cancer are associated with increased Src phosphorylation, mediating the β‐adrenergic/protein kinase A (PKA) axis, which enhances tumor cell migration, invasion, and growth.^203^ Norepinephrine also increases STAT3 phosphorylation, leading to elevated matrix metalloproteinases (MMP‐2 and MMP‐9), further driving ovarian cancer cell invasiveness. Similarly, in nasopharyngeal carcinoma, norepinephrine treatment elevates MMP‐2 and MMP‐9 levels, enhancing the invasiveness of the cancer cells.^204^ Adrenergic signaling activation is linked to EMT and increased invasiveness in several cancers, including ovarian,^205^ hepatocellular,^206^ breast,^207^ pancreatic,^83^, ^208^ and lung cancer cells.^209^ These findings highlight the critical role of neural signals in facilitating cancer progression through multiple mechanisms. Norepinephrine significantly increases levels of vascular endothelial growth factor (VEGF) in ovarian cancer,^210^, ^211^ melanoma,^212^ pancreatic cancer,^213^ oral cancer,^214^ and nasopharyngeal carcinoma.^215^ It can also stimulate angiogenesis in primary breast tissues by promoting VEGF signaling in TAMs.^85^ Similarly, norepinephrine signaling in lung cancer promotes angiogenesis indirectly by stimulating VEGF secretion from M2 TAMs.^216^ In prostate stroma, norepinephrine/β‐adrenergic signaling activates angiogenic switches, facilitating exponential tumor growth.^89^ Knockdown of Adrb2 (encoding the β2‐AR) reduces vascular density and inhibits prostate cancer progression in mice. Tumor‐associated lymphatic vessel density correlates closely with patient prognosis, and the sympathetic nervous system can influence lymphatic systems.^217^ In breast cancer mouse models, activation of β2‐ARs promotes VEGFC expression in cancer and stromal cells, increasing lymphatic vessel density and inducing stable expansion of lymphatic vessels draining the primary tumor. Conversely, blocking sympathetic nerves significantly (∼80%) inhibits lymphatic flow.^218^

### 3.2 Perineural infiltration by tumors

**FIGURE 4:**
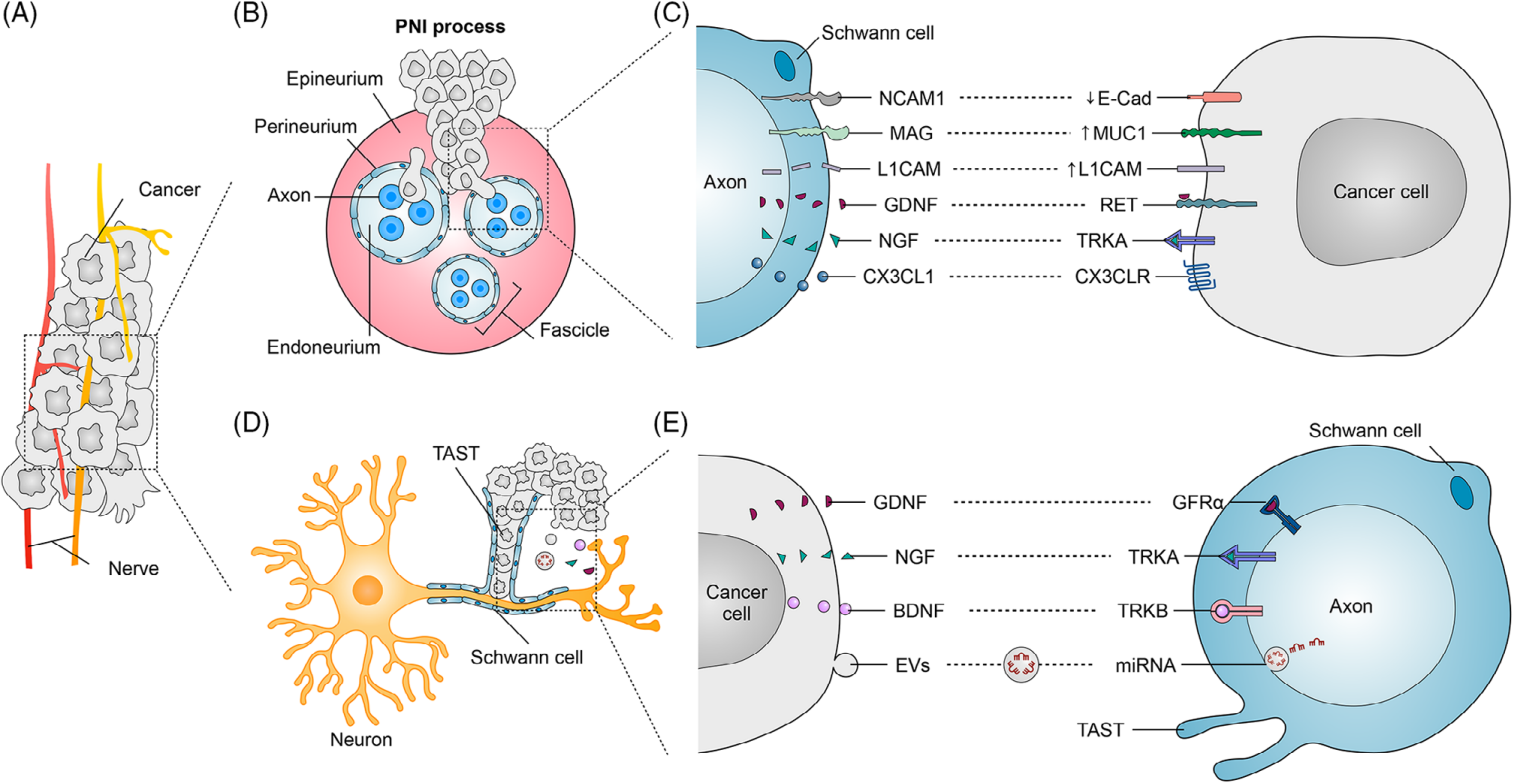
Perineural infiltration (PNI) and molecular interactions between Schwann cells and cancer cells. (A) Diagram shows how nerves interact with peripheral cancers. (B) A cross‐section of a nerve shows its protective layers—epineurium, perineurium, and endoneurium—surrounding the axons within a fascicle. The diagram illustrates how cancer cells infiltrate these layers during PNI. (C) Molecular signaling between Schwann cells and cancer cells. This section highlights the key molecular interactions driving communication between Schwann cells and cancer cells. Molecules such as neural cell adhesion molecule 1 (NCAM1), myelin‐associated glycoprotein (MAG), L1 cell adhesion molecule (L1CAM), glial cell line‐derived neurotrophic factor (GDNF), nerve growth factor (NGF), and fractalkine (CX3CL1) on Schwann cells interact with cancer cell receptors or ligands, including E‐cadherin (E‐Cad), Mucin 1 (MUC1), L1CAM, rearranged during transfection (RET), TRKA, and C‒X3‒C motif chemokine receptor 1 (CX3CR1). (D) The figure shows how cancer cells interact with Schwann cells and axons, highlighting the formation of the tumor‐activated Schwann cell tracks (TAST) by Schwann cells. (E) The figure illustrates the molecular crosstalk between cancer cells, Schwann cells, and axons. It highlights key signaling pathways through which cancer cells communicate with neurons. Cancer cells secrete growth factors such as GDNF, NGF, and brain‐derived neurotrophic factor (BDNF), which bind to their respective receptors (GFRα, TRKA, and TRKB) on Schwann cells and axons, promoting neuronal growth and cancer invasion. Additionally, cancer cells release extracellular vehicles (EVs) containing microRNAs (miRNAs), which contribute to neural reprogramming. The figure also showcases the TAST formed by Schwann cells, aiding in cancer progression.

PNI is a histopathological finding first identified in 1985, where malignant cells surround, invade, or reside within nerves, highlighting a key aspect of tumor‒nerve interactions (Figure 4A,B).^219^, ^220^ Clinically, PNI is an important factor in cancer management, often indicating the need for adjuvant radiotherapy and playing a role in the staging and risk stratification of various malignancies, including penile, head and neck, esophageal, and thyroid cancers. The incidence of PNI varies by cancer type, with rates reported as high as 98% in pancreatic cancer, 80% in head and neck squamous cell carcinoma, and 75% in prostate cancer, while colorectal cancer shows a lower incidence at around 33%.^221^ PNI can act as a conduit for tumor spread, allowing cancer cells to migrate along nerve fibers to distant sites, which contributes to tumor recurrence and metastasis. For instance, PNI is correlated with advanced clinical stage and metastasis in breast cancer.^15^, ^222^ In prostate and pancreatic cancers, PNI involving larger nerve diameters is associated with poorer prognosis.^223^, ^224^ Similarly, the depth of nerve invasion in colorectal cancer serves as an independent predictor of OS and disease‐free survival.^7^ As a result, PNI is recognized as an important prognostic factor in various cancers. The interaction between tumor cells and nerves during PNI is bidirectional and intricate, often involving the modulation of peripheral sensory nerve paracrine signals. This can lead to hypersensitivity, nerve sprouting, and further perineural invasion, highlighting the complex dynamics of tumor‒nerve crosstalk in cancer progression.^225^, ^226^, ^227^

Schwann cells, key glial cells in the PNS, surround nerve fibers and form myelin sheaths, playing a significant role in the TME (Figure 4C). These cells can interact directly with tumor cells via neural cell adhesion molecule 1, facilitating tumor cell invasion into nerves.^125^, ^228^ Schwann cells also release neurotrophic factors, such as GDNF, through paracrine signaling, which binds to the rearranged during transfection (RET) receptor on PDAC cells, promoting tumor cell migration toward neurons.^229^, ^230^ Additionally, Schwann cells secrete cell adhesion molecules and chemotactic factors, attracting tumor cells to nerves.^231^, ^232^ Schwann cells also play a role in modulating the immune response within tumors. For instance, they recruit TAMs to perineural invasion sites via C‒C motif chemokine ligand 2 (CCL2), which exacerbates nerve damage and tumor cell invasion.^233^ Recent research explores Schwann cells as potential therapeutic targets. By disrupting the signaling pathways between Schwann cells and tumor cells—such as blocking tumor‐promoting factors secreted by Schwann cells or interfering with their interactions with cancer cells—it may be possible to hinder tumor progression.^234^, ^235^ In summary, Schwann cells are increasingly recognized for their role in tumor progression, and ongoing research may reveal their potential as therapeutic targets, offering new avenues for anti‐cancer treatment strategies.^18^, ^236^

### 3.3 Tumor‐induced nerve remodeling

Tumor neurogenesis, or innervation, is a process where tumors recruit new axons and respond to nerve regeneration (Figure 4A,D), a hallmark of aggressive tumor behavior associated with poor prognosis.^10^, ^162^, ^237^ This phenomenon has been observed in various cancer types, including prostate and breast cancers.^12^, ^238^ Research shows that cancer cells express neurotrophic markers such as NGF, GDNF, and brain‐derived neurotrophic factor (BDNF), releasing axon‐guiding molecules that promote axonogenesis and attract nerves to the tumor site (Figure 4E). For instance, OSCC cells facilitate TME innervation by secreting NGF, which prompts nociceptive nerves to secrete CGRP and promote cancer growth.^239^ Similarly, nociceptive neurons can interact with melanoma cells, promoting synaptic growth and increasing responsiveness to harmful ligands and neuropeptides.^108^ Elevated levels of NGF precursors are strongly associated with neurogenesis in prostate cancer, where tumors can reprogram recruited nerves to develop into an adrenergic infiltrating phenotype that supports tumor growth and invasion.^240^ In OSCC, the loss of the tumor suppressor gene p53 has been shown to drive nerve reprogramming, reconnecting established nerves to an adrenergic phenotype through exosomes carrying microRNAs. These reprogrammed nerves provide growth‐promoting signals to the tumor and facilitate cancer cell invasion.^54^ Notably, PDAC cells reprogram proximal Schwann cells to become tumor‐activated cells, forming tumor‐activated Schwann cell tracks (TASTs), which create pathways for cancer cells to invade nerves.^241^ Peripheral tumors can also interact remotely with the brain by attracting neural progenitor cells, as seen in prostate and breast cancers. These progenitor cells cross the blood‒brain barrier, infiltrate the tumor, initiate neurogenesis, and differentiate into adrenergic neurons, further supporting tumor progression and metastasis.^242^ In summary, nerve reprogramming by tumors is a complex and critical process that drives cancer growth, invasion, and metastasis, representing an important area of research in tumor neuroscience.

## 4 NEUROIMMUNE CROSSTALK IN PERIPHERAL TUMORS

Neural signals modulate immune cell via adrenergic and cholinergic receptors, with norepinephrine binding to ARs on immune cells to influence cytokine production, cell migration, and immune function. This neural‒immune interaction is especially critical in the TME, where stress‐induced neural signals contribute to an immunosuppressive state, promoting tumor growth and metastasis. In turn, immune cells can influence neural structures by releasing cytokines and signaling molecules that affect nerve growth and function.

### 4.1 Stress and tumor immunology

**FIGURE 5:**
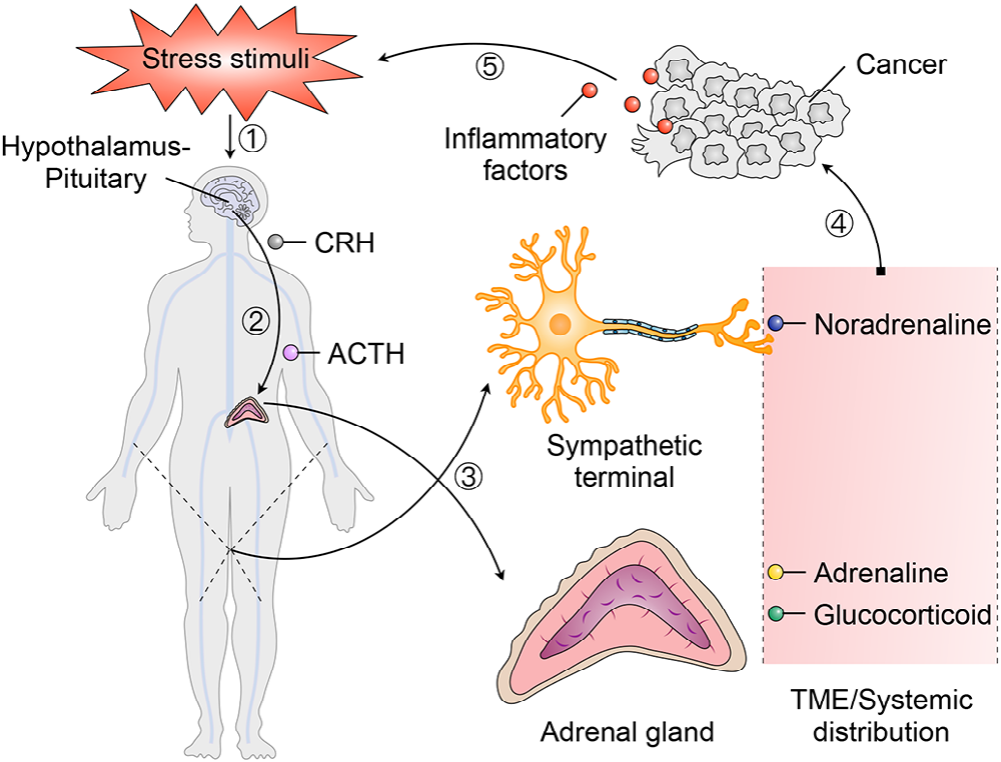
The stress response and its influence on the tumor microenvironment (TME). This schematic illustrates the biological pathway by which stress stimuli influence tumor progression via the hypothalamic‒pituitary‒adrenal (HPA) axis and the sympathetic nervous system. Upon exposure to stress, the hypothalamus releases corticotropin‐releasing hormone (CRH), which stimulates the anterior pituitary gland to secrete adrenocorticotropic hormone (ACTH) into the bloodstream. ACTH then prompts the adrenal glands to release adrenaline and glucocorticoids, which are distributed systemically, including to the TME. Simultaneously, the sympathetic nervous system is activated, releasing noradrenaline from sympathetic nerve terminals that innervate the tumor environment. These stress hormones, together with inflammatory factors, promote cancer cell survival, proliferation, and tumor progression by modulating the inflammatory conditions within the TME.

Stress is a physiological response aimed at restoring homeostasis, primarily through the activation of the sympathetic nervous system and the hypothalamic‒pituitary‒adrenal (HPA) axis.^243^ This activation triggers the release of adrenergic factors, such as norepinephrine and epinephrine, from sympathetic nerve endings and the adrenal medulla, along with glucocorticoids such as cortisol from the adrenal cortex.^244^, ^245^, ^246^ These hormones work together to coordinate the body's adaptive response to stress in tumor patients (Figure 5).

Cancer patients often experience peak stress during diagnosis, treatment, and relapse, which heightens the risk of anxiety and depression, potentially creating a synergistic interaction with the cancer itself.^247^, ^248^, ^249^ For instance, in breast cancer patients, elevated anxiety, stress, depressive symptoms, or increased nocturnal cortisol levels are linked to immune suppression mediated by tumor cells.^250^, ^251^ Tumors can elevate norepinephrine release within the TME, disrupt CNS and HPA axis activity, and intensify local and systemic inflammation, contributing to depression, sleep disturbances, and fatigue, thereby perpetuating a harmful cycle between stress and cancer.^252^, ^253^ Although the precise role of stress in cancer development is not fully understood, research suggests that stress promotes cancer progression by altering tumor characteristics, with animal studies and clinical observations confirming that adrenergic stress responses and inflammation play a significant role in this process.^86^, ^182^, ^254^ Recent animal studies provide strong evidence that stress accelerates cancer growth and metastasis by enhancing cancer markers, promoting immune evasion, and increasing inflammation, primarily through interactions with β‐adrenergic, prostaglandin, and glucocorticoid receptors.^255^, ^256^, ^257^, ^258^ Elevated stress in cancer patients has been shown to inhibit natural killer cell activity, reduce cytotoxicity, and promote tumor growth by inducing T helper (Th) cell differentiation and increasing the proportion of suppressive immune cells, such as MDSCs and Treg cells.^182^, ^259^, ^260^ Additionally, stress‐induced β‐adrenergic signaling increases cyclooxygenase‐2 (COX‐2) expression, prostaglandin secretion, and the release of pro‐inflammatory cytokines such as IL‐6, while also enhancing macrophage recruitment and M2 polarization in tumors.^261^ Social isolation is linked to increased M2 polarization in breast tumors, while higher depression levels are associated with increased norepinephrine in ovarian tumors.^262^ Both preclinical and clinical research have demonstrated that stress can reduce the efficacy of adjuvant and neoadjuvant cancer treatments, including chemotherapy, radiation therapy, and immunotherapy, through mechanisms mediated by glucocorticoids and/or catecholamines.^263^, ^264^, ^265^, ^266^ For instance, in mouse models of melanoma and lymphoma, social disruption stress or activation of β‐ARs has impaired CD8^+^ T‐cell responses and diminished the effectiveness of several immunotherapies.^267^, ^268^ Similarly, in models of breast, pancreatic, melanoma, colon, and lung cancers, stress‐induced β‐adrenergic signaling has been shown to negatively impact the success of programmed cell death‐1 (PD1)‐targeted immunotherapies.^269^, ^270^ Furthermore, in mouse models of gastric and breast cancer, β‐adrenergic activation has been shown to induce resistance to HER2‐targeted therapy with trastuzumab, with clinical observations supporting these findings by linking tumor expression of β‐ARs to reduced efficacy of trastuzumab in breast cancer patients.^271^, ^272^ These studies highlight the critical role of stress in undermining cancer treatment outcomes and the importance of managing stress as part of comprehensive cancer care.

### 4.2 Characterization of immune cells regulated by neural signals

**FIGURE 6:**
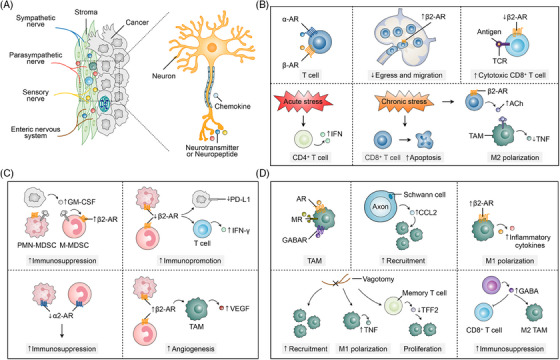
Neuro‐immune interactions in the tumor microenvironment (TME). (A) The diagram on the left shows sympathetic, parasympathetic, sensory, and enteric nerves infiltrating the tumor stroma. These neurons release neurotransmitters and neuropeptides, which interact with cancer cells and immune cells via chemokine signaling, influencing both tumor progression and immune responses. (B) T cells express both α‐ and β‐adrenergic receptors (β‐ARs), and their activation triggers distinct immune responses. Increased β2‐AR signaling impairs T‐cell egress and migration from lymphoid organs, limiting their ability to reach tumor sites. Conversely, the downregulation of β2‐AR on CD8^+^ T cells enhances their cytotoxic functions and improves antigen recognition through interactions with T‐cell receptor (TCR). Acute stress can boost immune responses by promoting interferon (IFN) production in CD4^+^ T cells, while chronic stress has the opposite effect, inducing apoptosis in CD8^+^ T cells and weakening immune surveillance, thereby promoting tumor progression. Moreover, chronic stress and β2‐AR activation increase acetylcholine (ACh) production, which drives M2 polarization of tumor‐associated macrophages (TAMs) and suppresses tumor necrosis factor (TNF) production, creating an immunosuppressive tumor microenvironment. (C) β2‐AR signaling in myeloid‐derived suppressor cells (MDSCs) enhances their immunosuppressive activity by increasing granulocyte‒macrophage colony‐stimulating factor (GM‐CSF) production. Conversely, reduced β2‐AR signaling lowers programmed death‐ligand 1 (PD‐L1) expression and boosts IFN‐γ production in T cells, leading to enhanced immune activation. Additionally, α2‐AR signaling in MDSCs further strengthens their immunosuppressive effects, promoting tumor immune evasion. In TAMs, β2‐AR activation supports angiogenesis by increasing vascular endothelial growth factor (VEGF) secretion, which facilitates tumor growth. (D) AR, MR, and GABAR on TAMs regulate their activation and function in the tumor microenvironment. Schwann cells, in conjunction with axons, release CCL2 to recruit TAMs to the tumor site, increasing immune cell infiltration. β2‐AR signaling in TAMs elevates the production of inflammatory cytokines, promoting M1 polarization and a pro‐inflammatory response. Vagotomy enhances TAM recruitment and M1 polarization by boosting TNF production, thereby supporting an anti‐tumor immune response. It also reduces TFF2 expression in memory T cells, which boosts their proliferation. Conversely, GABA production by CD8^+^ T cells promotes TAM polarization towards the M2 phenotype, leading to immunosuppression in the TME.

Crosstalk between immune cells, cancer cells, and nerves can impact cancer progression (Figure 6A). For instance, neurotransmitters can have immunomodulatory effects that influence protumor inflammation, anti‐tumor immunity, and immunosuppression. Immune cells present in the TME, including T cells, MDSCs, TAMs, B cells, neutrophils, and dendritic cells, participate in neural signaling regulation.

#### 4.2.1 T cells

The functional stages of T cells—antigen recognition, activation, clonal expansion, effector functions, and memory formation—enable them to identify and eliminate pathogens while maintaining immune balance.^273^, ^274^, ^275^ In the TME, T cells express β‐ARs (primarily β2‐AR), responding to neural signals, with β‐adrenergic signaling playing a key role in regulating anti‐tumor immune responses (Figure 6B).^276^, ^277^ The sympathetic nervous system influences the lymphatic system, responsible for transporting lymphocytes, by β2‐AR signaling in lymph nodes, which inhibits T‐cell outflow and reduces lymphocyte infiltration in tissues.^278^ Conversely, β‐AR agonists rapidly suppress CD8^+^ T‐cell movement in tissues and temporarily inhibit CD4^+^ T‐cell movement, suggesting that sympathetic nervous system stimulation can impair leukocyte mobility and potentially weaken immune responses.^279^ 6OHDA‐mediated sympathectomy enhanced CD8^+^ T‐cell responses to antigens in mice, discovering that this enhanced response was mediated by β2‐AR, rather than β1‐AR or α‐AR.^280^ Exhausted CD8^+^ T cells express β1‐AR in response to stress‐associated catecholamines, inhibiting their function and accumulating around sympathetic nerves, but blocking β1‐adrenergic signaling can slow T‐cell exhaustion, enhance anti‐tumor function, and synergistically promote effector T‐cell responses with ICIs therapy.^29^ Acute stress activates ARs on CD4^+^ T cells and B cells, increasing IgG1 and interferon (IFN) production, while chronic stress promotes apoptosis of these cells.^281^ Similarly, β2‐AR signaling knockdown increased the proportion of effector CD8^+^ T cells while decreasing the percentage of regulatory CD4^+^ T cells in the TME.^269^ Daher et al. reported that β2‐AR signaling strongly inhibited the initial activation of effector CD8^+^ T‐cell responses, suggesting it suppresses the activation of naïve CD8^+^ T cells.^267^ In the spleen, T cells expressing β2‐AR produce ACh when stimulated by sympathetic innervation.^282^ Under stress conditions, Ach produced by T cells inhibits tumor necrosis factor (TNF) production in TAMs that express nicotinic ACh receptors. Additionally, autonomic innervation has a direct impact on the recruitment and fate of immune cells within the TME.^283^, ^284^

#### 4.2.2 MDSCs

MDSCs, a class of immature immune cells including monocytic (M‐MDSCs) and granulocytic (PMN‐MDSCs) subsets, originate from bone marrow precursors and play a crucial role in the TME (Figure 6C).^285^, ^286^ Upon recruitment to the TME, MDSCs secrete various immunosuppressive factors, such as nitric oxide, reactive oxygen species, transforming growth factor‐beta, and IL‐10, which collectively inhibit T‐cell activity and function.^287^, ^288^, ^289^ MDSCs also express β‐ARs, and the secretion of granulocyte‒macrophage colony‐stimulating factor by cancer cells leads to increased expression of β2‐AR on MDSCs, further promoting tumor‐induced immunosuppression.^290^, ^291^, ^292^ Activation of β2‐AR on MDSCs downregulates glycolysis and enhances fatty acid oxidation, thereby augmenting the immunosuppressive function of MDSCs.^293^ Research indicates that knocking down β2‐AR in MDSCs reverses their immunosuppressive function, enhancing T‐cell proliferation and IFN‐γ production, while knocking down Adrb2 slows tumor growth, reduces programmed death‐ligand 1 (PD‐L1) expression, and lowers serum levels of immunosuppressive cytokines.^292^ β2‐AR positively regulates adenylate cyclase activity and cAMP levels, which is related to the mechanism of the anti‐tumor immune response. In contrast, α2‐AR exerts opposite regulatory effects on cAMP and downstream signals to those of β‐AR. Disruption of α2‐AR signaling leads to the accumulation of MDSCs, which suppress anti‐tumor immunity and promote tumor growth.^294^, ^295^ MDSCs also influence the maturation and function of dendritic cells and promote the generation of TAMs, affecting antigen presentation and immune responses.^296^ Additionally, MDSCs can directly secrete VEGF to support tumor vascularization.^297^, ^298^ β2‐AR signaling has been found to increase the accumulation of MDSCs in the TME and promote tumor angiogenesis.^216^ In lung cancer, adrenergic signaling indirectly stimulates angiogenesis by promoting VEGF secretion from M2 macrophages.^216^

#### 4.2.3 Macrophages

TAMs originate from peripheral blood monocytes and differentiate into macrophages upon entering the TME, where their surface is equipped with receptors that respond to neural signals, including adrenergic, cholinergic, and GABA receptors (Figure 6D).^299^, ^300^, ^301^ Under stress conditions, increased adrenergic signaling and β2‐AR agonist administration lead to an increase in TAMs in breast cancer, while β2‐AR antagonists inhibit their recruitment and metastasis.^302^ In prostate and pancreatic cancers, TAMs increase as the cancer progresses, and this increase is nerve dependent. Additionally, vagotomy may accelerate the onset of pancreatic cancer and promote tumor growth by recruiting TAMs and mediating inflammation.^303^, ^304^ Vagus nerve stimulation activates postsynaptic adrenergic nerves in the celiac ganglion and inhibits TNF release from TAMs, while vagotomy removes this immunosuppression, resulting in increased TNF levels.^126^, ^127^, ^305^, ^306^ Bilateral vagotomy reduces the expression of trefoil factor 2 (TFF2) in the spleen of mice, suggesting that cholinergic signaling enhances TFF2 release, which, in turn, inhibits the expansion of MDSCs and suppresses inflammation and colon cancer development.^307^ GABA signaling can also produce anti‐inflammatory and immunosuppressive effects by directly inhibiting macrophage function.^308^ GABA derived from cancer cells can activate GABA‐BR, suppressing the expression of CCL4 and CCL5, thus inhibiting the tumor infiltration of T cells and dendritic cells.^309^, ^310^ Additionally, in colon cancer, B cells can produce GABA, which limits the anti‐tumor response by inhibiting CD8^+^ T‐cell cytotoxicity and promoting the survival of M2‐type TAMs.^311^

#### 4.2.4 Interactions between immune cells and nerves

Immune cells, including T cells, MDSCs, and TAMs, express neural receptors on their surfaces, enabling them to receive neural signals. Interestingly, these immune cells can also affect neural structures, a dynamic area of research. For instance, in Alzheimer's disease, conditioned media from T cells treated with photobiomodulation therapy has been found to promote neural stem cell differentiation.^312^ Macrophages have a complex role in neurogenesis. Brain macrophages produce essential factors for the migration of new neurons, yet inflammation mediated by macrophages can be detrimental to neurogenesis and the survival of new neurons.^313^ However, similar studies exploring this interaction within peripheral TME remain limited.

### 4.3 Neuroinflammatory mediators and their roles

In peripheral tumor neuroscience, the interplay between neuroinflammation and tumor progression is crucial, as tumor‐associated inflammation significantly influences the development, growth, and treatment response of peripheral tumors, with various key mediators and cells playing essential roles in this process. M2‐TAMs are central to the inflammatory response in peripheral tumors, secreting cytokines, growth factors, and enzymes that promote tumor growth, angiogenesis, and metastasis, while also contributing to a tumor‐supportive microenvironment by remodeling the extracellular matrix and suppressing anti‐tumor immune responses. Moreover, while innate immune cells such as neutrophils and B cells have garnered significant attention in cancer research, their roles in tumor‒nerve interactions remain less understood. In the TME, tumor‐associated neutrophils are recruited by signals primarily from macrophages, as well as tumor and stromal cells, and are generally classified into two types: anti‐tumor (N1) and pro‐tumor (N2).^314^, ^315^, ^316^ Neutrophils are closely linked to stress and inflammation as key mediators of the body's rapid immune response.^317^ Neutrophils are also recruited into the TME by neuropeptides and in response to DA, which induces apoptosis and inhibits their functions.^318^, ^319^ Additionally, tumor cells can promote neutrophil infiltration into the perihippocampal meninges via the CCR2‒CCL2 axis, contributing to cognitive decline and anorexia.^320^ In high‐grade gliomas, increased neuronal infiltration and neutrophil extracellular trap (NET) formation, identified as an oncogenic marker, promote glioma cell proliferation, migration, and invasion.^321^ HMGB1 binds to NF3 and activates the NF‐κB pathway, leading to IL‐10 (IL‐8) secretion, which recruits neutrophils and induces further NET formation via the PI3K/AKT/ROS axis, while targeting NET formation or IL‐8 secretion inhibits glioma progression.^321^ These findings suggest that neutrophils play a crucial role in tumor‒nerve interactions; however, while current research has primarily focused on brain tumors, their higher enrichment in peripheral tissues warrants further investigation. Moreover, B cells can express nerve‐related receptors, such as ACh receptors, and secrete neurotropic factors such as GABA, while also interacting with other immune cells, including T cells and macrophages, to shape the tumor's immune response.^311^, ^322^, ^323^ These complex interactions between neutrophils, B cells, other immune cells, and neural cells complicate tumor‒nerve crosstalk, highlighting the need for further investigation into the roles of neutrophils and B cells in cancer.

In summary, neuroinflammatory mediators and inflammation‐associated cells play complex and diverse roles in peripheral tumors. Macrophages, neutrophils, cytokines, chemokines, and prostaglandins significantly influence the TME, affecting tumor growth, metastasis, and treatment outcomes. A deeper understanding of these interactions is essential for developing advanced therapeutic strategies in peripheral tumor neuroscience.

## 5 THERAPEUTIC IMPLICATIONS AND STRATEGIES

The primary goal of translating cancer neuroscience research into clinical practice is to develop more effective treatments, with the complex interactions between cancer‒nerve‒immune driving several clinical trials aimed at improving therapeutic outcomes (see Table 1). Clinically, neuromodulatory drugs, such as β‐adrenergic agents and DA and glutamate receptor modulators, have shown anti‐cancer potential, with β‐blockers, in particular, being associated with improved tumor prognosis. Although these strategies have been tested in small prospective trials, large‐scale randomized controlled trials are still needed to verify their clinical efficacy. Drugs targeting neural paracrine signaling are less commonly used, although targeting neural activators in these pathways may prove effectiveness; however, genetic alterations in many cancers complicate this approach. Repurposing existing drugs offers a faster pathway to cancer treatment due to their known safety profiles, but the lack of tumor specificity and potential side effects remain significant challenges for neuromodulatory therapies.

**TABLE 1:** Clinical trials investigating inhibitors of nerve‒cancer interactions in peripheral cancers.

Cancer type	Therapeutic targets	Intervention	Trials no.
Prostate cancer	β‐AR	Carvedilol	NCT02944201
Advanced melanoma	β‐AR, opioid receptor, and immune checkpoints	Propranolol, naltrexone, and ICIs	NCT05968690
Hepatocellular carcinoma	β‐AR	Propranolol and carvedilol	NCT06233708
Malignant soft tissue sarcoma	β‐AR and chemotherapy	Propranolol and doxorubicin	NCT03108300
Pancreatic, hepatocellular, and biliary tract cancer	β‐AR, immune checkpoints, and chemotherapy	Propranolol, ICIs, and paclitaxel	NCT05451043
PDAC	β‐AR	Propranolol	NCT06145074
Breast cancer	β‐AR	Carvedilol	NCT02177175 NCT01724450
Breast cancer and melanoma	β‐AR	Propranolol	NCT02013492
Prostate cancer	β‐AR	Propranolol	NCT03152786
Cancer bone metastases	NGF signaling	Tanezumab	NCT02609828
PDAC	Acetylcholine receptor	Bethanechol	NCT03572283
Prostate cancer	Denervation	Botulinum toxin	NCT01520441

Abbreviations: β‐AR, β‐adrenergic receptor; ICI, immune checkpoint inhibitor; NGF, nerve growth factor; PDAC, pancreatic ductal adenocarcinoma.

*Source*: https://clinicaltrials.gov/.

### 5.1 Targeting tumor‒nerve interactions

Given the diverse mechanisms of tumor‒nerve crosstalk and their clinical and pathological significance, this knowledge can be translated into several key intervention strategies: inhibiting tumor growth, overcoming therapeutic resistance and tumor adaptation to stress, enhancing anti‐tumor immunity, and preventing metastasis.^324^

Neural signaling plays a significant role in tumor biology by modulating both adrenergic and cholinergic pathways. Pharmacological interventions using selective and non‐selective β‐blockers (e.g., propranolol, metoprolol) and parasympathomimetics (e.g., bethanechol) have demonstrated potential as adjuvant cancer therapies.^325^ Notably, differences in cholinergic responses have been observed between various cancers, such as gastric and pancreatic cancers. Retrospective studies suggest improved outcomes in cancer patients who use β‐blockers.^326^ Clinical trials such as NCT02944201, NCT0383802, NCT03572283, and NCT04245644 are investigating these drugs in both non‐metastatic and metastatic cancers. β‐AR antagonists have been found to enhance the therapeutic effects of chemotherapy and immunotherapy. Adrenergic signaling has been implicated in chemotherapy resistance, with evidence suggesting that the addition of β‐adrenergic antagonists can improve chemotherapy outcomes. For instance, norepinephrine‐induced overexpression of DUSP1 in ovarian cancer cells increases apoptosis, indicating that adrenergic signaling may reduce chemotherapy sensitivity.^26^ In a mouse model of pancreatic cancer, cold stress was shown to activate sympathetic nerve responses and β‐AR, leading to increased resistance to cisplatin and paclitaxel.^27^ Similarly, in triple‐negative breast cancer, β‐AR antagonists combined with anthracycline chemotherapy reduced metastasis and improved chemotherapy efficacy. β1‐AR antagonists, such as nebivolol, have also been shown to inhibit the growth of colon and breast cancers by suppressing oxidative phosphorylation in cancer cells and preventing endothelial cell proliferation, thereby reducing tumor angiogenesis.^31^, ^327^ β‐ARs signaling plays a crucial role in chemoresistance in cancer, and blockade of ARs can act as a chemosensitizer in combination with treatment for breast cancer,^23^ lung cancer,^28^, ^34^ colorectal cancer,^328^, ^329^, ^330^ cervical cancer,^331^ uveal melanoma,^332^ and multiple myeloma.^24^ In addition, a β1‐AR blocker (nebivolol) specifically impeded oxidative phosphorylation in cancer cells, preventing tumor angiogenesis by blocking endothelial cell proliferation and limiting colon and breast cancer growth.^25^ Overall, β‐ARs appear to hold promise in improving cancer‐related biomarkers, and large‐scale randomized controlled trials are currently underway to assess the long‐term efficacy of these drugs for managing cancer‐related stress responses.

### 5.2 Modulating neuroimmune crosstalk

Immune checkpoint blockade is now a standard cancer treatment, with studies showing that combining neuroactive drugs with ICIs can modulate the TME. Research has demonstrated that β‐AR blockade enhances immunotherapy efficacy by regulating immune responses, and preclinical studies across various cancers suggest that inhibiting adrenergic signaling can improve anti‐tumor immune effects. Propranolol co‐administered with an anti‐CTLA‐4 (cytotoxic T‐lymphocyte associated protein 4) antibody improved ICB efficacy in patients with soft tissue sarcoma.^30^ Nine patients with metastatic melanoma treated with a combination of propranolol and pembrolizumab, an anti‐PD1 checkpoint inhibitor, achieved an objective response rate of 78%, with elevated IFN‐γ and reduced IL‐6.^38^ C‒X‒C motif chemokine receptor 4 (CXCR4) is a promising target for anti‐cancer therapy, and it has been found that β2‐AR can bind to CXCR4 and that CXCR4‒β2AR heteromers are present in human cancer cells, which might help to develop targeted therapeutics for the treatment of CXCR4‐positive cancer drugs.^333^ ICIs show limited efficacy in immunologically cold tumors, partly due to the absence of CD8^+^ T cells near malignant cells.^334^, ^335^ Combining radiation therapy with β‐ARs blockade has the potential to enhance the response in tumors that are resistant to immunotherapy alone, such as pancreatic and prostate cancers. Immune dysfunction is linked to prolonged antigen exposure, impairing the effector function of CD8^+^ T cells. Research indicates that β1‐ARs, rather than β2, play a significant role in immune failure. Inhibiting β1‐AR activity enhances CD8^+^ T‐cell function in melanoma and helps prevent T‐cell exhaustion.^29^ Advances in multi‐omics technology and artificial intelligence (AI) have enabled multi‐scale mapping of nerve‒cancer‒immune interactions, offering insights into the specific role of nerves in tumor immune regulation. Future research should prioritize exploring the regulatory effects of neural signals on immune cell phenotypes within the TME. These findings could enhance current immunotherapies and support the development of novel treatment strategies.

### 5.3 Emerging therapies

Strategies to inhibit neuroregeneration focus on the complex interplay between tumors and the nervous system, as tumor cells exploit regenerative mechanisms to support their growth and metastasis.^336^ Under normal conditions, the nervous system's regenerative capacity can facilitate tumor expansion, particularly in cancers such as pancreatic and breast cancer, which stimulate nerve growth to create a favorable microenvironment.^241^, ^337^ Inhibiting neuroregeneration typically targets growth factors, such as NGF, and key signaling pathways such as MAPK and PI3K/Akt.^338^ Developing drugs or immunotherapies that target these elements can help prevent nerve regeneration, thereby restricting tumor growth and spread. Unlike surgical or chemical neurolysis, targeting neurotrophic factors involved in tumor axonogenesis may offer a more effective approach, as it preserves the essential neural support within organs without damaging existing structures. While neurotrophic factors such as NGF are crucial during peripheral nerve development, they play a minimal role in maintaining neuronal function in adults. Instead, NGF primarily mediates pain by activating the tropomyosin receptor kinase A (TRKA) receptor in sensory neurons.^339^, ^340^ Studies have shown that systemic administration of anti‐NGF antibodies does not significantly affect neuronal or cognitive functions, making NGF an attractive therapeutic target for preventing nerve growth within the TME.^341^, ^342^ Anti‐NGF antibodies, TRKA inhibitors, and NGF‐targeted siRNA delivered via nanoparticles have shown promise in reducing cancer growth and metastasis in animal models, and these approaches are advancing to clinical trials. Beyond inhibiting tumor axonogenesis, anti‐NGF antibodies also offer the benefit of alleviating cancer‐related pain, demonstrating their dual potential as effective cancer therapies.^343^

## 6 CHALLENGES AND FUTURE DIRECTIONS

Research in peripheral tumor neuroscience is uncovering the intricate connections between the nervous system and tumor progression. Sensory and sympathetic nerves within the PNS interact with tumor and immune cells through the release of specific signaling molecules, influencing tumor growth and metastasis. These peripheral nerves also play a role in critical tumor‐related processes such as angiogenesis, immune evasion, and metabolic regulation. As this field evolves, researchers are exploring novel treatment strategies, such as blocking nerve‒tumor cell signaling or employing neuroregulation techniques to inhibit tumor growth. Additionally, applying neuroscience tools to study the interactions between the nervous system and tumors is revealing new therapeutic targets. This growing understanding holds the potential for more personalized and effective cancer treatments, as novel interventions may be tailored to disrupt the neural mechanisms driving tumor development.

### 6.1 Research gaps and limitations

Surgical denervation has demonstrated tumor‐suppressing effects in mouse models of prostate and gastric cancer, but translating this approach to clinical practice presents significant challenges. Complete denervation may lead to severe side effects, limiting its broad application. The complexity of the TME and the intricate nerve innervation of different organs complicate the implementation of this strategy. Additionally, complete denervation can result in postoperative discomfort and potentially cause organ dysfunction.^344^ While denervation has shown efficacy in experimental settings, its potential side effects must be carefully considered in clinical practice. Future research should prioritize optimizing denervation techniques to develop safer and more effective methods. Minimally invasive surgery, focusing on local nerve resection, could offer a viable option, particularly in cases where direct tumor resection poses high risks. This approach may help reduce surgical complications while inhibiting cancer progression. Alternatively, local injection of chemical agents or drugs targeting nerves involved in tumor growth offers a less invasive solution. For example, botulinum toxin (BoNT‐A) has been used to induce autonomic denervation, with clinical trials showing that it can trigger apoptosis in prostate cancer cells. This method holds promise for reducing the side effects of traditional surgery and could become a valuable tool in cancer treatment.^345^

Mouse cancer models fail to fully capture the complexity of human diseases, particularly in cancer neuroscience. Future research must prioritize the development of animal models that better simulate the neural invasion characteristics seen in human cancers. Genetically modified and humanized patient‐derived xenograft models can induce neural invasion in mice that more closely resembles human pathology.^346^, ^347^, ^348^ Additionally, using species with anatomical and physiological similarities to humans, such as pigs, may provide a more accurate platform for studying human pathophysiology. While various neural signaling inhibitors have shown promise in preclinical studies, these pathways are not only involved in tumor growth but are also critical for normal neural and bodily functions. This makes it challenging to develop therapies that target nerve‒cancer interactions without compromising normal neural function. Furthermore, the effectiveness of cancer treatments depends on disease stage and patient‐specific responses to neural signaling inhibitors. Identifying predictive biomarkers for tailored therapies will enhance treatment selection and advance personalized neuro‐oncology care.^325^

### 6.2 Personalized medicine approaches

Research demonstrates that a patient's psychological state significantly influences the effectiveness of tumor treatments. Stress hormones (e.g., norepinephrine, epinephrine, glucocorticoids) may negatively impact both adjuvant and neoadjuvant therapies, although this issue has been insufficiently addressed in clinical studies. Synthetic glucocorticoids, such as dexamethasone, are commonly used in cancer treatment to alleviate chemotherapy‐induced nausea, improve lymphoma chemotherapy outcomes, and reduce inflammation during immunotherapy.^349^, ^350^ However, these drugs may diminish the efficacy of certain adjuvant therapies and, in some cases, promote cancer progression.^351^, ^352^, ^353^ For instance, in non‐small cell lung cancer, glucocorticoids have been linked to lower response rates to ICIs and shorter survival times. Psychological interventions can mitigate stress, preventing adverse effects from medications, particularly for patients with contraindications. Stress management strategies are recommended during critical treatment phases, such as the perioperative and adjuvant treatment periods, to assess their effectiveness compared to other time points. Additionally, in vitro studies typically co‐culture neurons with cancer cells, overlooking other crucial components of the TME. More advanced 3D organoid culture methods, which incorporate nerve cells, glial cells, immune cells, and cancer‐associated fibroblasts, offer a more accurate representation of the in vivo tumor environment.^354^, ^355^, ^356^ Ongoing clinical trials with patient‐derived organoids may lead to personalized neuro‐targeted therapies by identifying an individual's unique “neuro‐molecular signature.”

### 6.3 Collaborative and multidisciplinary research

Researchers utilize advanced high‐resolution imaging techniques, including electron microscopy, two‐photon and three‐photon microscopy, and multiphoton laser scanning microscopy, to investigate the complex interactions between neurons and cancer cells.^357^, ^358^, ^359^ These technologies allow for detailed observation of neural networks within the TME, as well as the overall tumor architecture. Positron emission tomography is particularly valuable for tracking cholinergic neural activity to assess tumor spread, especially in prostate cancer.^360^ These imaging methods aid in selecting candidates for neuromodulatory therapies. Further advancements, such as time‐lapse microscopy, fluorescence molecular tomography, and in vivo microscopy, deepen our understanding of the interactions between neurons, cancer cells, and glial cells.^361^, ^362^ High‐resolution microscopy, including electron and atomic force microscopy, reveals intricate details of neuronal processes, glial cell extensions, and their interactions with cancer cells.^362^ Techniques such as light‐sheet microscopy and tissue clearing enable the generation of 3D neuroanatomical maps of tumors and organs, which provide valuable insights for refining local tumor control strategies.^363^, ^364^

Single‐cell RNA sequencing offers a detailed view of neurotransmitter receptor gene expression, shedding light on synaptic connections between neurons and glioma cells and clarifying the molecular mechanisms that link cancer and the nervous system.^365^ Spatial transcriptomics further identifies novel therapeutic targets, overcoming the limitations of bulk tissue analyses that may obscure specific neural subtypes.^366^, ^367^, ^368^ Multi‐omics sequencing technologies allow for the identification of cell‐specific molecular changes, providing a deeper understanding of neuron‒cancer interactions. AI and machine learning have become crucial tools in studying these interactions, accelerating data analysis, pattern recognition, and integrating mathematical modeling with AI to drive advancements in tumor neuroscience. Collaboration between tumor researchers and oncologists, alongside the sharing of technologies and models, is essential for promoting an interdisciplinary approach to precision medicine. Ultimately, this approach—especially when applied to large patient cohorts—aims to improve treatment outcomes in cancer patients.

## AUTHOR CONTRIBUTIONS

**Xin‐Hua Liang and Ya‐Ling Tang**: designed the study. **Hua‐Yang Fan**: drafted the manuscript. **Xin‐Hua Liang and Ya‐Ling Tang**: revised the manuscript. All authors have approved the submitted version of the manuscript.

## CONFLICT OF INTEREST STATEMENT

The authors declare they have no conflicts of interest.

## ETHICS STATEMENT

Not applicable.

## Data Availability

Not applicable.
